# Menstrual Health Under Constraint: A Meta-Synthesis of Refugee Women’s Experiences

**DOI:** 10.3390/healthcare14131974

**Published:** 2026-07-02

**Authors:** Francesca Marchetti, Fabiana Staccioli, Margaret Smith, Francesco Rasi, Francesca Zambri, Sofia Colaceci

**Affiliations:** 1 Departmental Faculty of Medicine, Saint Camillus International University of Health and Medical Sciences (UniCamillus), 00131 Rome, Italy; stacciolifabiana@gmail.com (F.S.); francesco.rasi@unicamillus.org (F.R.); sofia.colaceci@unicamillus.org (S.C.); 2 Departmental Faculty of Medicine and Surgery, Azienda Ospedaliera San Camillo Forlanini, 00152 Rome, Italy; 3 National Centre for Disease Prevention and Health Promotion, Italian National Institute of Health, 00161 Rome, Italy; francesca.zambri@iss.it

**Keywords:** menstrual health, refugee women and girls, menstrual equity, menstrual hygiene management, qualitative meta-synthesis

## Abstract

**Highlights:**

**What are the main findings?**

- Menstrual health among refugee women and girls is shaped by the interplay of structural barriers, socio-cultural norms, and displacement-related conditions that limit safe and appropriate management.
- Women and girls adopt coping strategies within constrained conditions, often relying on informal support networks but at a cost to health and dignity.

**What are the implications of the main findings?**

- Effective responses require integrated, multisectoral, and gender-transformative approaches that address structural inequalities and stigma.
- Strengthening menstrual health literacy and community engagement may support more equitable and sustainable interventions, with midwives playing a key role in supporting and guiding change.

**Abstract:**

**Background/Objectives**: Refugee populations face significant barriers in accessing healthcare services, particularly in sexual and reproductive health (SRH), with important implications for menstrual health. Limited access to adequate menstrual products, safe sanitation facilities, and appropriate information contributes to period poverty among migrant and refugee women, exacerbating conditions of vulnerability and discrimination. The present study aims to explore the lived experiences, barriers, and facilitating factors in the management of menstrual health and menarche among refugee women and girls. **Methods:** A systematic meta-synthesis was carried out between January and May 2026 using the PubMed, Cochrane Library, Scopus and LILACS databases and grey literature sources. The SPIDER framework was applied to guide the research question and search strategy. Qualitative and mixed-method primary studies and grey literature reports containing qualitative findings describing experiences of menstruation among refugee women and girls were included. Studies published in English, Italian, Spanish, and Portuguese were eligible. Study quality was appraised using the Joanna Briggs Institute (JBI) critical appraisal checklist. Data were analysed using a thematic synthesis approach as described by Thomas and Harden. **Results:** A total of 24 studies were included. Six analytical themes were identified: (1) structural constraints affecting access to resources and services; (2) context-dependent menstrual management practices; (3) female support networks; (4) menstruation as a socially constructed and learned experience; (5) constrained agency and compromised dignity under conditions of stigma; and (6) physical and psychological impacts. Overall, menstrual health was shaped by the interaction of structural barriers and socio-cultural norms, which limited safe and dignified management. Significant gaps in knowledge and preparedness were observed, particularly prior to menarche. Women and girls relied on coping strategies and informal support networks despite associated trade-offs for health and dignity. **Conclusions:** Menstrual health among refugee women and girls is shaped by structural inequalities, socio-cultural norms, and conditions of displacement. Addressing these challenges requires integrated, multisectoral approaches that go beyond product provision to tackle underlying determinants. Strengthening menstrual health literacy, engaging communities, and supporting the role of healthcare professionals such as midwives is essential to support more equitable and sustainable menstrual health interventions. Findings should be interpreted in light of the heterogeneity of study contexts and methodological quality.

## 1. Introduction

Global migration and forced displacement represent major humanitarian challenges, with the number of forcibly displaced individuals reaching historically high levels worldwide [1]. According to the United Nations High Commissioner for Refugees (UNHCR), an estimated 117.3 million people worldwide were living in situations of forced displaced as of mid-2025, including refugees, asylum seekers, internally displaced persons (IDPs), and other populations in need of international protection [1]. Women and girls account for approximately 51% of the global forcibly displaced population, corresponding to more than 60 million individuals affected by conflict, persecution, violence, and displacement [2].

Access to sexual and reproductive health (SRH) services is often limited in humanitarian settings, where refugee women and girls face significant barriers to care, including legal, economic, and sociocultural barriers [3,4]. In this context, refugee women and girls often face marked disparities in SRH outcomes, while menstrual health remains an overlooked component of SRH, despite its importance for health, dignity, and gender equality.

Contemporary perspectives on menstrual health emphasize that menstruation extends beyond menstrual hygiene management and should be understood as a multidimensional phenomenon shaped by the interaction of material, social, cultural, and environmental factors [5,6]. From a social determinants of health perspective, menstrual health is influenced by broader structural and contextual factors. Within this framework, access to menstrual products, adequate sanitation facilities, accurate information, and supportive social environments shapes how women and girls experience menstruation and its implications for health, well-being, dignity, and social participation [7,8].

Sexual and reproductive health and rights are recognised as fundamental human rights and essential components of Universal Health Coverage (UHC). International guidance, including the Sphere Handbook [9], increasingly recognises menstrual health as an essential component of humanitarian response and SRH services. Accordingly, menstrual health is increasingly recognised as a determinant of health, well-being, and social participation within international policies and frameworks [10,11]. Nevertheless, substantial inequalities in access to and utilisation of SRH services persist among migrant and refugee women, highlighting a gap between international commitments and the realities experienced in humanitarian settings [3]. In these contexts, menstrual needs are frequently deprioritised relative to competing health and survival needs, which contributes to “period poverty,” a multidimensional condition characterised by limited access to menstrual products, safe sanitation, and adequate information [5,7].

Numerous studies report that migrant and refugee women and girls experience substantial barriers in managing menstruation across the migration journey. These include limited access to essential resources and infrastructure, as well as difficulties in maintaining privacy and hygiene practices [5,12,13]. Displacement disrupts social networks, reduces autonomy, and limits engagement with healthcare services, which are often perceived as inaccessible or culturally inappropriate [14,15]. These challenges are further exacerbated by overcrowding, resource scarcity, and adverse environmental conditions, which can further compromise menstrual health and broader SRH outcomes [16]. Addressing menstrual health in humanitarian settings requires multidisciplinary collaboration between healthcare professionals, including midwives and community health workers, alongside Water, Sanitation, and Hygiene (WASH) actors; educators; social workers; and policymakers [17,18].

Structural barriers experienced by migrant and refugee women and girls manifest in discomfort, shame, and reduced participation in daily activities [5]. Adolescents frequently report inadequate preparation for menarche, insufficient knowledge, and limited access to culturally appropriate information [19,20,21]. Sociocultural taboos surrounding menstruation further restrict communication and reinforce stigma, shaping menstrual practices and access to care [22,23].

These intersecting determinants highlight the need to better understand how menstrual health is experienced within refugee populations. Qualitative evidence on the lived experiences of migrant and refugee women and girls remains fragmented, despite the growing recognition of menstrual health as a public health priority. Expanding the evidence base on menstrual health among refugee women and girls may support progress towards Sustainable Development Goals (SDG) 3 and 5, which emphasise equitable access to health services and the reduction in gender-based health inequalities [24].

This meta-synthesis aims to systematically synthesise qualitative evidence on the lived experiences, barriers, and facilitators of menstrual health and menarche among refugee women and girls. Specifically, the objectives are to: (1) explore experiences of menstruation and menarche among refugee women and girls; (2) identify structural, sociocultural, and individual barriers and facilitators influencing menstrual health management (MHM); (3) synthesise qualitative evidence to inform more equitable and context-sensitive menstrual health interventions in humanitarian settings.

## 2. Materials and Methods

### 2.1. Study Design

This study employed a meta-synthesis design to integrate qualitative research exploring the experiences of refugee women and girls. Reporting followed the Enhanced Transparency in Reporting the Synthesis of Qualitative Research (ENTREQ) statement [25] and the Preferred Reporting Items for Systematic Reviews and Meta-Analyses (PRISMA) guidelines [26] (Supplementary Materials Tables S1 and S2). The review was retrospectively registered in INPLASY (registration number INPLASY202650040; https://doi.org/10.37766/inplasy2026.5.0040). Some methodological refinements, including modifications to the search strategy and eligibility criteria, were introduced during the conduct of the review to improve its comprehensiveness and methodological appropriateness. A qualitative review approach was adopted, based on secondary synthesis of qualitative findings, aimed at identifying recurring patterns and underlying themes.

### 2.2. Search Strategy

A systematic search was conducted in MEDLINE (PubMed), Scopus, the Cochrane Library and LILACS databases between January and May 2026. In the same period, grey literature searches were performed in the ReliefWeb repository and on the websites of major international organisations and humanitarian agencies working with refugee populations, including UNHCR; United Nations International Children’s Emergency Fund (UNICEF); World Health Organization (WHO); WHO/UNICEF Joint Monitoring Programme for Water Supply, Sanitation and Hygiene (JMP); United Nations Population Fund (UNFPA); International Organization for Migration (IOM); Médecins Sans Frontières (MSF); Save the Children; Oxford Committee for Famine Relief (OXFAM); the International Rescue Committee (IRC); and Cooperative for Assistance and Relief Everywhere (CARE) International. The research strategy was developed using the SPIDER (Sample, Phenomenon of Interest, Design, Evaluation, and Research type) [27] framework. For each element of the SPIDER framework, a list of keywords was developed (Appendix A, Table A1), supplemented with synonyms, lexical variants, and controlled vocabulary terms from database thesauri. Search terms were combined using the Boolean operator OR within each concept, and AND between concepts, to maximise search sensitivity (Appendix A, Table A2 and Table A3).

### 2.3. Inclusion and Exclusion Criteria

Studies conducted in migration, transit, or reception settings involving women and girls who were refugees, IDPs, asylum seekers and undocumented migrants were included. Eligibility was restricted to studies with a primary focus on menstruation, menarche, or menstrual health. Qualitative or mixed-method primary studies published in peer-reviewed journals, as well as relevant grey literature reports containing qualitative findings, and written in Italian, English, Spanish or Portuguese, were included. The language restriction was applied to ensure accurate screening, data extraction, and interpretation of qualitative findings based on the language competencies of the research team.

Studies that included populations of “stable” migrants (e.g., long-term foreign residents, second-generation migrants, or international students) or mixed samples that did not allow disaggregation of refugee or migrant status were excluded. Studies addressing other sexual and reproductive health topics unrelated to menstruation or menarche (e.g., gynaecological cancers, pregnancy or female genital mutilation), quantitative studies, as well as secondary and tertiary literature were also excluded.

### 2.4. Study Selection Process

**Figure 1:**
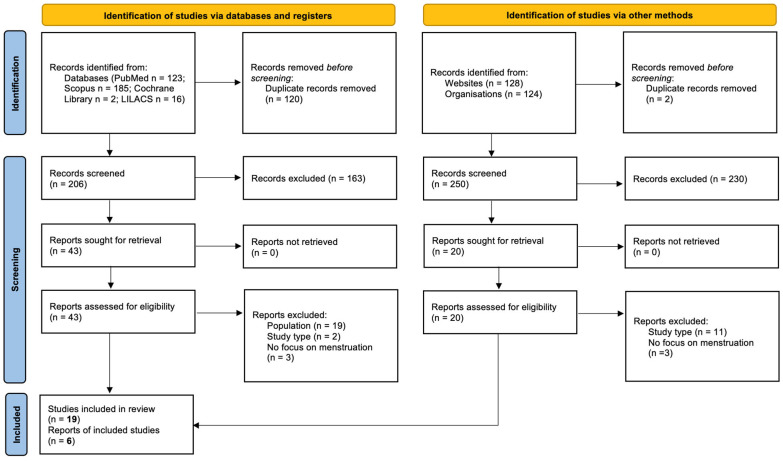
Study selection process (PRISMA flow diagram) [26].

The study selection process is illustrated in a PRISMA flow diagram (Figure 1). After searching the selected databases, all retrieved records were imported into Rayyan [28], where duplicates were removed. Title and abstract screening was conducted independently by two reviewers (F.M., F.S.) in blind mode, based on the predefined inclusion and exclusion criteria. The full texts of potentially eligible studies were then retrieved and assessed independently by the same reviewers for final inclusion. In addition, studies addressing broader aspects of sexual and reproductive health were considered eligible if they reported substantial and analytically relevant qualitative data pertaining to menstrual experiences (e.g., themes, subthemes, or participant quotations). Any disagreements were resolved through discussion or, when consensus could not be reached, by a third reviewer (S.C.).

The methodological quality of the included studies was independently assessed by two reviewers (F.M., F.S.) using the Joanna Briggs Institute (JBI) critical appraisal checklist for qualitative research [29] in order to assess the credibility, dependability, and transparency of the findings. Quality assessment was used to inform the synthesis process and the interpretation of findings rather than as a criterion for weighting evidence. All studies contributed to the synthesis; however, particular attention was paid to whether analytical themes were supported across studies with varying methodological quality. The final themes were predominantly informed by studies assessed as moderate-to-high quality. Disagreements were resolved by discussion or, when necessary, through consultation with a third reviewer (S.C.). One study was excluded due to insufficient methodological quality.

### 2.5. Data Extraction and Analysis

Data were extracted from the results section of the included studies. Potential overlap in samples and settings across studies was assessed and transparently documented to minimise duplication bias. When potential overlap was identified, studies were compared in terms of setting, participant characteristics, study aims, and the nature of the qualitative findings reported. Studies were retained when they addressed different research questions and contributed complementary qualitative insights relevant to the review objectives, thereby preserving the complexity and breadth of the evidence base. Moreover, the potential influence of overlapping samples on the interpretation of findings was considered during the synthesis. Participant quotations and authors’ interpretations were independently analysed by two reviewers (F.M., F.S.) using a thematic synthesis approach [30], informed by the principles of thematic analysis described by Braun and Clarke [31]. A thematic synthesis approach was adopted for its suitability in integrating rich qualitative data across diverse contexts, enabling the development of descriptive and analytical themes and the identification of cross-cutting patterns relevant to public health.

The analysis was conducted following a predominantly inductive approach. Following familiarisation with the data, line-by-line coding was conducted to identify units of meaning. Codes were then discussed between reviewers until consensus was reached and subsequently organised into descriptive themes through an iterative process of comparison and refinement. In a further stage of analysis, descriptive themes were interpreted and synthesised into higher-order analytical themes, allowing a more conceptual understanding of the data. Reflexivity was considered throughout the analytic process, with researchers acknowledging and critically reflecting on how their perspectives and assumptions could influence data interpretation. An audit trail was maintained throughout the analytic process to ensure transparency and rigour, including documentation of coding decisions, theme development, and iterative modifications made during the synthesis.

### 2.6. Ethical Considerations

Although this study did not involve direct interaction with participants, ethical considerations informed the review process. Particular attention was paid to the respectful representation of refugee women and girls’ experiences and to the use of inclusive and culturally sensitive language.

Reflexivity was considered throughout the synthesis process. The background of the research team, including expertise in midwifery, public health, qualitative research, and vulnerable populations, informed data interpretation and thematic development.

## 3. Results

### 3.1. Review Identification and Selection

Database and grey literature searches yielded 326 and 252 records, respectively. After removal of duplicates and title and abstract screening, full-text articles were assessed for eligibility, of which 25 met the inclusion criteria. Following methodological quality appraisal, one study (Turkmen Sanduvac et al., 2017 [32]) was excluded due to insufficient methodological quality. A total of 24 records (19 studies and 5 reports) were therefore included in the meta-synthesis (Figure 1), published between 1988 and 2026.

All included studies explored women’s experiences, needs, or perceptions related to menstruation. The studies were conducted across multiple regions, including Europe [33,34], Africa [16,35,36,37,38,39,40,41], Asia [13,21,42,43,44,45,46,47,48,49,50,51,52], and North America [53], and encompassed diverse humanitarian settings and sociocultural contexts. Publication years ranged from 1988 to 2026, reflecting changes over time in humanitarian responses, sociopolitical conditions, and awareness of menstrual health.

All studies employed qualitative designs, with some incorporating mixed-methods approaches. Data collection methods were largely consistent across studies and primarily included focus groups and semi-structured or in-depth interviews (Table 1). The total sample comprised predominantly refugee and internally displaced (ID) women and adolescent girls across diverse humanitarian settings. Participants ranged from early adolescence to older adulthood (approximately 13 to over 50 years) and represented varied vulnerability profiles. In particular, one study included an adolescent girl with a disability [49], while another explored the menstrual experiences of lesbian, gay, bisexual, trans and intersex (LGBTI) individuals in humanitarian settings [52].

The studies by Ivanova et al. [37] and Kemigisha et al. [38] appeared to involve partially overlapping samples and study settings. Nevertheless, both were retained, as they addressed different research objectives and contributed complementary qualitative findings relevant to the review question. Particular attention was paid during data extraction and synthesis to minimise potential duplication and overrepresentation of findings.

### 3.2. Quality Appraisal

The methodological quality of all included studies was rated as high, with scores ranging from 5 to 10 (Table 2). Overall, the aims and research methodologies were appropriate across studies, and data collection procedures were adequately described. Ethical considerations were addressed in most cases, and many studies demonstrated a rigorous approach to data analysis with clear presentation of findings.

However, some methodological limitations were identified. In particular, recruitment strategies were not clearly reported in several studies [13,16,33,34,41,42,48,49,50,51,52], and researcher reflexivity was often insufficiently addressed, with limited discussion of researchers’ roles, potential biases, and influence on the research process [16,21,39,42,44,47,48,49,50,51,52]. The grey literature reports included in the synthesis generally demonstrated lower methodological quality, mainly due to limited reporting of methodological procedures and reflexivity, although they provided relevant contextual insights into menstrual health experiences in humanitarian settings. One report (Turkmen Sanduvac et al., 2017 [32]) scoring 1/10 was excluded from the synthesis due to insufficient methodological quality.

Despite these limitations, the overall methodological quality was considered adequate to support the synthesis. The final analytical themes were informed by studies with varying methodological quality and were predominantly supported by studies assessed as moderate-to-high quality. Findings derived primarily from individual grey literature reports were incorporated within existing themes and interpreted cautiously.

### 3.3. Thematic Synthesis Findings

Thematic synthesis identified six analytical themes (Table 3 and Table 4) describing menstrual health and menarche experiences among refugee women and girls: (1) structural constraints shaping access to menstrual health resources and services; (2) embodied and context-dependent menstrual management practices; (3) female support networks as key resources in menstrual management; (4) menstruation as a socially constructed and learned experience; (5) constrained agency and compromised dignity under conditions of menstrual stigma and exposure; and (6) physical and psychological impacts of menstrual health challenges. These themes indicate that menstrual health is a multidimensional phenomenon shaped by the interaction of structural conditions, social norms, and lived experiences.

**Theme 1: Structural constraints shaping access to menstrual health resources and services**

Across studies, menstrual health management was shaped by structural constraints, including infrastructural and environmental conditions, economic hardship, and limited access to healthcare services.

***Theme 1.1: Environmental and infrastructural barriers***

Women and girls reported inconsistent access to clean water, which was described as essential for daily survival and hygiene practices: *“Without water you can’t do anything. Somebody will say ‘I rather have water in the house even though I don’t have food’ because we all know water is life”* [10]. Combined with poor sanitation conditions—often unhygienic, distant, overcrowded, and damaged—women’s ability to manage menstruation safely and privately was hindered. At night, inadequate lighting further contributed to unsafe environments and increased the risk of violence. One participant shared: *“In the night hours, I will fear to go there when there is no light because the latrine is a little far so, I fear if someone is to come and rape me”* [10]. Women also adopted adaptive strategies to avoid using sanitation facilities during the most crowded or unsafe times: *“We use plastic buckets when the latrines are too far or too dirty. We try to empty them at night to avoid being seen”* [32]. In one report, inaccessible sanitation facilities were described as creating additional challenges for menstrual management for a girl with a disability [49]. Similarly, in another study, a trans heterosexual man reported difficulties in identifying an appropriate and private space to manage menstruation, stating: *“I went into the men’s bathroom in a park. There were urinals only. There was no place with a door and a lock”* [52]. This account illustrates additional menstrual management challenges related to the accessibility and inclusivity of sanitation facilities.

***Theme 1.2: Economic constraints and limited access to menstrual and hygiene re-sources***

Economic constraints further limited access to menstrual hygiene products, with participants frequently describing financial hardship and limited product availability. Although some distribution systems for menstrual products were reported, often through non-governmental organizations (NGOs), these were described as insufficient or inconsistent: *“We are rarely provided with menstrual pads and soap, and not all girls and women in the camp receive them”* [25]. Participants often described having to prioritise competing basic needs, such as food or children’s needs, over menstrual hygiene products. In situations of extreme deprivation, menstrual products were perceived as secondary to immediate survival needs: *“A bread bundle is worth a thousand pads”* [49]. One participant described using menstrual pads as a valuable form of exchange to obtain money to purchase medication for her ill brother*: “I could sell the menstrual pads that I had received. I had to raise the amount of money we needed”* [25]. In a few cases, access through local markets was possible, indicating variability in availability across settings.

***Theme 1.3: Access to and quality of healthcare services for menstrual health***

Access to healthcare services was also limited by multiple factors, including lack of awareness of available services and systemic inadequacies, as reflected in participants’ accounts: *“I don’t know whether there is a health centre here inside the camp*” [25], “*There’s no relief because there’s no medical care*” [35]. Participants described difficulties in accessing appropriate treatment, shortages of medications, and overburdened healthcare systems. The presence of male staff and a lack of trust in the services also led to reluctance to seek care.

Overall, these findings show that menstrual health is embedded within broader structural inequalities, with environmental, economic, and healthcare barriers jointly restricting access to essential resources and services.

**Theme 2: Embodied and context-dependent menstrual management practices**

Menstrual management emerged as a dynamic, context-dependent process shaped by available resources and ongoing adaptation. Limited access to menstrual products frequently led women and girls to adopt adaptive strategies, including the use of improvised materials to collect menstrual blood: “*I tear clothes and use them”* [27]*, “I use diapers or toilet paper*” [35], and *“… if we do not have those [old clothes] we pick them up from the rubbish and wash them*” [30]. When menstrual products were available, participants reported using a range of menstrual materials, including disposable pads, reusable cloth pads, and, in one study, menstrual underwear. Disposable pads were often perceived as convenient because they reduced the burden associated with washing reusable materials: *“[…] do not have a lot of time to be washing [reusable cotton pads]. It is better for me to use pads and throw them in the garbage*” [24]. However, some women and adolescent girls expressed concerns about the poor quality of available disposable products and their associated physical consequences (see also Theme 6). Other participants preferred reusable pads because of their absorbency and cost-effectiveness, while some described combining reusable and disposable products as the most suitable strategy: *“Currently, with the use of reusable pads, I use the money I save to buy disposable pads of better quality”* [48]. Other menstrual products, such as tampons and menstrual cups, were mentioned only in one report. A cisgender lesbian woman described these products as unacceptable, stating: *“I do not want anything to penetrate my body”* [52].

Daily management practices, including changing, washing, drying, and disposing of menstrual materials, were particularly burdensome for girls and women due to shortages of water, soap, and adequate facilities: “*We need soap to wash our pads, but we have none!*” [25]. The lack of private spaces also posed challenges, particularly in drying menstrual materials, which increased their visibility within the community: *“… but the problem is how and where can you hang it up to dry?*” [25]. To avoid exposure, one participant explained: “*The only chance we get to wash our menstrual pads is during the night*” [25].

These practices were shaped by the contextual challenges women and girls faced in their environments. Some participants describe nighttime as particularly challenging for menstrual hygiene management, due to the distance to latrines, lack of lighting, and lack of privacy: “*When menstruation happens at night, we suffer*” [25], “*We cannot change the pads inside our home, because it is dark inside and if we try to search for a pad […] everybody will be awakened and asked, ‘What is going on with you?’ So, we prefer to keep silent and wait until morning*” [25]. Another challenging circumstance was managing menstruation during transit, as shared by one participant: “*Before this time [before war and displacement], I was eager to see it every month. However, when menstruation appeared while I was traveling [during displacement], I hated myself for being a female*” [25].

Beyond ongoing menstrual management, participants reflected on their initial experiences of menstruation, which played a key role in shaping subsequent practices. Many participants reported being unprepared for menarche, often experiencing fear or confusion: “*I was scared and flustered. I was afraid I was sick or something*” [33]. In most cases, mothers were identified as the first point of contact when menarche occurred. Their presence was described as a source of information and emotional support: “*She told me: ‘You are blossoming [zahharti]’, which means you are a grown-up, and then she explained [about menstruation]*” [34]; “*My mom said: ‘Why are you scared?’ […]. She told me that what happened is normal*” [33]; “*My mother said, ‘I’ll bring you a sanitary pad.’ Naturally, I put it on incorrectly [laughs]. My mother placed it for me and informed me about it*” [34]. In other cases, some mothers were “*Too embarrassed to discuss these matters*” [33], or girls feared telling them what happened, limiting the sharing of knowledge about menstruation.

Together, these findings show that menstrual management is an adaptive and context-dependent practice for refugee women and girls, shaped by structural constraints and continuously negotiated within changing environments.

**Theme 3: Female support networks as a key resource in menstrual management**

In the absence of adequate institutional support, informal networks of women were a crucial resource for menstrual management. Participants frequently relied on mothers or, alternatively, on female relatives (sisters, aunts, grandmothers, cousins) and peers for practical assistance, emotional support, and knowledge sharing: “*I can only talk about this with women, and we can share and talk about our period and what we use and how we wash, but only with women and only with women my age*” [24].

Moreover, women and girls established support networks that supported menstruation management through sharing and solidarity. This fostered a sense of belonging and mutual support, helping girls feel less alone in the face of difficulties: “*But I am happy with it because all my friends, my mother, my sister, my aunts, we are all the same. So, I don’t feel like it’s only me*” [27], “*We try to help each other as sisters, because no one else will*” [32].

Such networks often compensated for gaps in formal systems, underscoring the role of relational and community-based support in menstrual health management among displaced populations. Overall, informal female networks functioned as key resources for menstrual health management in contexts of structural neglect.

**Theme 4: Menstruation as a socially constructed and learned experience**

Participants’ menstrual knowledge and perceptions were shaped through socialisation and cultural transmission within families, peers, and community environments.

***Theme 4.1: Transmission of knowledge and knowledge gaps***

Participants reported varying levels of knowledge, with many describing significant gaps in understanding the origin of menstrual blood and the physiological processes underlying menstruation: “*…[menstruation] was like a miracle to me because I did not know anything about it*” [28]. In many cases, information about menstruation was only provided to girls after menarche and was often superficial, incomplete, or limited to religious and social norms that girls were expected to follow: “I *[a mother to her daughter] just told her about how long the period lasts, that it comes every month”* [33]*; “I [a mother to her daughter] just told her […] that during Ramadan she should break her fast [during the few days she is on her period] and make up for these days later”* [33]. Participants also described their menstrual education as shaped by fear and restrictions, including prohibitions during menstruation related to leaving the home, hygiene practices, consuming certain foods or beverages, and interacting with men, often under the threat of severe consequences: *“No, avoid boys whenever you have periods. […] Tell them not to come when you are on your periods. If they touch you, you’re going to get pregnant”* [26].

However, the included studies also reported occasional instances of adequate menstrual knowledge levels of the participants.

Knowledge of menstruation and menstrual management practices was primarily transmitted through informal channels, including mothers, family members, and peers: “*She [the mother] then explained to me everything and asked me to let her know in case the blood continues to come, so that she provides for me what to use*” [28]; “*… our appa [elder sister] was tackling that topic of menstruation and telling us how you take care of ourselves*” [26].

Schools and teachers represented another source of information on menstruation through teacher–student interaction; in some cases, the topic was included in school curricula as part of biology or sex education classes. Even in these cases, the information was often incomplete and superficial: “*[The biology teacher] explained a little. He said that when girls [reach puberty], they become mature. Girls grow armpit hair, boys start to have deep voice, girls get their period, boys … I forgot what he said*” [33]. In some cases, educational intervention improved understanding of the menstrual biology. As one participant explained, following a teacher’s advice, she spoke openly with her family to challenge certain misconceptions about menstruation: “*My family is ignorant and holds erroneous beliefs. They discuss such things at home [that showering is harmful], but after we read a book assigned by the teacher, I returned home and informed them of the proper information*” [34].

The participants cited other sources of knowledge on menstruation, including NGO-led educational programs, religious places (mosques and madrassa schools), and Scouts.

***Theme 4.2: Cultural beliefs and practices about menstruation***

Many of the included studies reported widespread menstrual taboos. Participants described a cultural context in which menstruation was expected to be concealed from society, particularly from men, and where open discussion is discouraged: “*How can we ask for information in front of all the family members here?”* [25]; “*Our parents have taught us that it is not acceptable to talk about it [menstruation], or that it is wrong*” [33]. Across different settings, menstruation was associated with notions of shame and social unacceptability. Ghandour et al. [34] reported that participants and other people in the camps used the term “aib” (ﻋﻴﺐ) to refer to menstruation, a concept associated with something inappropriate, unacceptable, and impolite, and often perceived as embarrassing, shameful, or taboo. Similarly, Majed & Touma [49] described how, during a focus group discussion (FGD), an older woman reacted with visible discomfort when an adolescent girl openly referred to menstrual blood, gently kicking her in the leg and saying *“Aayb!”* (“shameful”). This observation suggests that menstrual stigma may also be reinforced through non-verbal responses and social regulation of menstrual discussions. Such norms may limit the transmission of accurate knowledge and reinforce fears of exposure and social judgment.

Cultural beliefs and practices further shaped menstrual experiences and menstrual behaviours. These included misconceptions and myths, social norms, and behavioural restrictions during menstruation (e.g., abstaining from sexual intercourse, not fasting, or praying while menstruating): “*Grandma stated: ‘If you shower when you first get your period, this is the end; there will be no childbearing, sexual organs contract, and then it [menstruation] ceases, and you are no longer menstruating*” [34]; “*My mother and grandmother forbid me and ask me to stay away from the day-to-day activities. I am not allowed to drink too much water and not to eat sour types of food. I have to sleep far from our family members, and I don’t like it*” [37]; *“When I’m menstruating, we can’t hold Qur’an, I can’t go to the masjid. I cannot even fast. It is a sin!”* [51].

Some participants reported negative perceptions of menstrual blood, which they described as “toxic” [36], “*bad*, *rotten and filthy*” [34], alongside negative emotions related to bleeding. At the same time, many women and girls expressed positive perceptions of menstruation, viewing it as having a purifying and protecting role: *“[Menstruation] It absorbs sickness from the body. Consider my grandma; she is elderly and no longer menstruates, and as a result, she suffers from various diseases. For us, it safeguards our bodies against disease and removes poisons*” [34].

Despite the strong influence of taboos and prejudices, in a few isolated cases, girls and women described menstruation as a normal and healthy phenomenon: “*There is nothing embarrassing about it because every girl will have her period, not only us*” [36].

Overall, menstruation emerged as a socially constructed experience, shaped by the interaction between knowledge transmission, cultural beliefs, and social norms.

**Theme 5: Constrained agency and compromised dignity under conditions of menstrual stigma and exposure**

Menstrual stigma and social exposure were pervasive across the included studies, significantly affecting women’s experiences and behaviours. Participants described fear of being seen while managing menstruation (e.g., changing pads, washing, drying, purchasing, and disposing menstrual materials), anxiety about leakage, and efforts to conceal menstruation, reflecting social pressures associated with menstrual visibility: “*I don’t even leave the tent because I’m terrified someone will notice [the menstruation]*” [35]; “*We have to hide the damp menstrual cloths underneath existing clothing or mattresses to dry*” [37]; “*Some girls hide it. I mean, they get their period… but they do not tell anyone*” [33]. Feelings of embarrassment also extended to obtaining and disposing of menstrual products. Participants described discomfort when purchasing menstrual materials from male vendors, including *“If a man is selling, I feel ashamed and ask for diapers”* [51] and *“You have to wait for every other customer to leave to buy a pad…”* [52], and adopted discreet disposal practices to avoid social exposure and menstrual stigma: *“Why burn them? Because if dogs started messing around the rubbish bags, everybody will know who had her period, and this is haram [shameful]”* [49].

Poor living conditions, characterised by environmental and infrastructural constraints, overcrowded housing, and non-gender-segregated latrines, led to lack of privacy and forced exposure: “*Every time one of us gets our period, it feels like the entire camp knows*” [35], “*In our culture, menstruation is private; however, now it’s exposed for everyone to witness*” [35]. This contributed to experiences of shame and social judgment, reinforcing stigma and limiting women’s ability to manage menstruation with dignity, as one participant shared: *“… It feels as if our dignity has been stripped away*” [35].

These conditions often resulted in restricted agency, with participants reporting limited freedom of action, lack of control over their circumstances, and the need to endure discomfort without choice: “*We do not have the freedom to do as we wish*” [25]; “*It’s humiliating and engenders a sensation of having lost all control over our lives*” [35]. In some situations, participants described being at the mercy of events, with nothing to do but wait and endure: “*When menstruation happens at night, […] we cannot go to the toilet because it is too dark, and the toilet is far away. […] We can’t do anything until it is dawn, but we pray for the night to be shorter so we can change our menstrual pads*” [25]; “*We’re compelled to endure in silence*” [35].

Perceptions that menstrual health was not prioritised further exacerbated these experiences, contributing to feelings of neglect and marginalisation: “*I feel as if my health isn’t regarded as a priority*” [35], “*We’re all in this together, but it ought not to be this way*” [35]. In some cases, menstrual-related challenges also limited participation in daily activities, including education, as one participant described: “*All the days I have it, I don’t come to school*” [28].

These findings show how stigma and structural conditions intersect to constrain agency and undermine dignity in menstrual management.

**Theme 6: Physical and psychological impacts of menstrual health challenges**

Menstrual health was experienced as both a physical and psychological burden. Participants reported a range of physical health issues, including irregular menstrual cycles and menstrual pain: “*Before the war, my cycle was predictable. Now, it comes twice a month or not at all*” [35]; “*I have FGM [female genital mutilation] and when I get my period it’s super pain! Sometimes I start crying, crying until I get sleep*” [23]. Beyond these physiological symptoms, inadequate hygiene conditions and limited access to appropriate menstrual products further contributed to adverse health outcomes. The use of damp or dirty cloths caused skin rashes, itching, general discomfort, and in some cases, vaginal and urinary tract infections: “*I have to cut old fabric into pieces, however, they complain about rashes and itching*” [35]; “*All of my girls have developed rashes and infections because we can’t afford proper pads or clean water*” [35].

These physical experiences were closely intertwined with emotional and psychological distress, reflecting the cumulative burden of physical discomfort, social stigma, and environmental constraints. Feelings of confusion, anxiety, shame, and fear were commonly reported in connection with menarche, social exposure, and threats to personal safety, reflecting the combined impact of physical discomfort, social stigma, and environmental constraints: “*Menstruation has become a nightmare*” [32] and “*This is a topic I am embarrassed to talk about; I will never ask anybody*” [34].

Gender-related distress was also reported, with some participants expressing negative feelings associated with menstruation and its implications for their identity and daily life. In one case, menstruation was described within broader gendered beliefs that associated women with impurity and men with cleanliness: *“Men are part of the ‘clean class’ and women and girls are part of the ‘dirty class’ because they have periods and men do not. Men are pure like flowers”* [50]. Some women and girls viewed menstruation—or even being born female—as a curse, expressing rejecting their condition: “*We suffer a lot because of menstruation, and we always curse the day we were born*” [25]; “*I hated myself for being a female*” [25]; “*You know what I wish every time my menstruation occurs here in the camp…? I wish I had never been born, or that I had been born male!”* [25]. In the UNFPA report [52], LGBTI participants reported experiences reflecting dominant gendered expectations linking menstruation to femininity and reproductive capacity. One participant recalled being told that *“Having a period means that I am a ‘woman’”* [52], while another stated that *“Bleeding means that the person with bleeding is fit for pregnancy”* [52]. However, one trans heterosexual man challenged these assumptions, stating: *“If someone menstruates, it is not something to diminish their masculinity. It is natural.”* [52].

Together, these findings suggest that menstrual health is deeply embodied, with physical and emotional dimensions closely interconnected and shaped by broader social and structural factors.

## 4. Discussion

**Figure 2:**
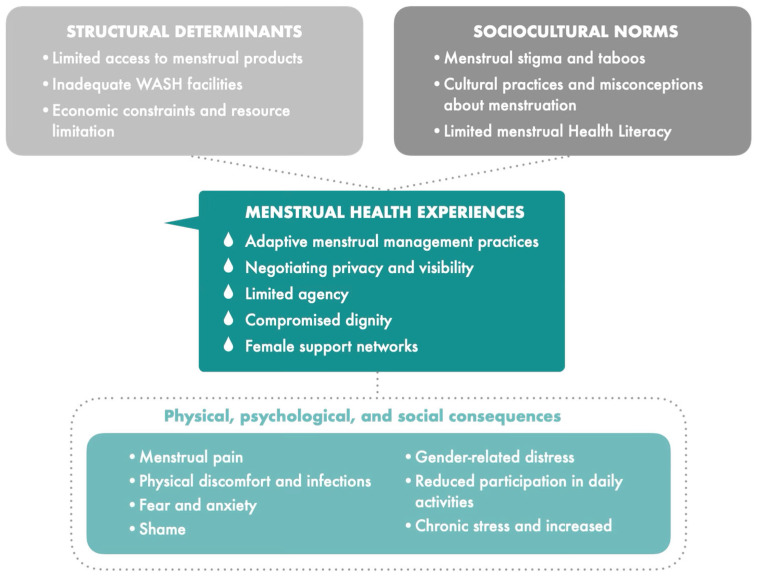
Conceptual figure summarising the relationship between structural barriers, sociocultural norms, and consequences for menstrual health.

This meta-synthesis explores the lived experiences, barriers, and facilitating factors related to menstrual health and menarche among refugee women and girls. Despite variation in humanitarian contexts and study populations, the analysis identified interconnected themes that revealed common patterns of menstrual health experiences. The findings indicate that menstrual health is shaped by the interaction of structural barriers and sociocultural norms, which together influence menstrual experiences and their physical, psychological, and social consequences (Figure 2).

Menstrual health was strongly influenced by structural determinants, including inadequate WASH facilities, economic constraints, and limited access to healthcare services. Consistent with previous research [18,54,55,56], these factors interacted to create cumulative barriers that restricted women’s ability to manage menstruation safely, privately, and with dignity. These findings suggest that menstrual health challenges are rooted in broader systemic inequities that affect access to resources and opportunities [5,57]. Beyond these structural barriers, the findings underscore the central role of stigma and sociocultural norms in shaping menstrual experiences. Menstruation was frequently associated with secrecy, shame, and the need for concealment, particularly in relation to male family members and the wider community [58]. This reflects the “menstrual concealment imperative” described by Wood [59], whereby menstruation is socially constructed as something that must remain hidden in order to conform to gendered norms. In the contexts examined, structural constraints such as lack of privacy, overcrowded living conditions, and forced exposure further intensified this imperative, illustrating how menstrual stigma is not only culturally constructed but also materially reinforced.

Limited menstrual health literacy, characterized by knowledge gaps and misconceptions surrounding menstruation, menarche, and menstrual blood, shaped menstrual experiences, particularly among adolescent girls. Consistent with previous research [54,60,61,62], the findings highlight the family environment as a key setting for the intergenerational transmission of menstrual knowledge. Family may serve as sources of support and normalization, but they can also perpetuate misconceptions, restrictive practices, and menstrual stigma. In turn, stigma and taboos surrounding menstruation can limit open dialogue and knowledge sharing [60], reducing access to comprehensive sexual and reproductive health information and undermining menstrual preparedness and effective menstrual management [63]. The findings further suggest that younger generations may challenge inherited norms and adopt more open and positive attitudes towards menstruation.

Despite pervasive barriers, women and girls were not passive recipients of structural and sociocultural constraints. Instead, they actively negotiated menstrual management through a range of coping strategies, including the use of improvised materials, adaptation of practices, and reliance on informal support networks. These strategies were often shaped by competing priorities, as women and girls frequently had to balance menstrual needs against other essential demands, such as food security, healthcare, and family responsibilities. These adaptations may therefore be understood as forms of constrained agency, reflecting resilience and efforts to preserve dignity, privacy, and social acceptability within highly constrained environments, while simultaneously involving trade-offs and limited opportunities for genuine choice or empowerment [5,60]. The findings also highlight the importance of female and peer support networks as sources of practical and emotional support. Consistent with previous studies [54,64], these networks may represent collective forms of resilience that partially mitigate the impact of structural and sociocultural challenges surrounding menstruation.

The findings further suggest that menstrual health is experienced as an embodied phenomenon in which physical symptoms, emotional distress, stigma, and social constraints are closely interconnected. Experiences of menstrual pain, infections, fear of social exposure, shame, and gender-related distress illustrate how menstrual challenges extend beyond biological processes and are embedded within the social and emotional dimensions of daily life. This interconnected burden may be further understood through the lens of chronic stress. Refugee women and girls are exposed to multiple and persistent stressors, including displacement, insecurity, poverty, and limited access to basic services [65,66]. In this context, the concept of allostatic load may provide a useful framework for interpreting these findings. Allostatic load refers to the cumulative physiological burden associated with chronic stress exposure [67]. Repeated exposure to overlapping stressors may contribute to adverse sexual and reproductive health outcomes [68,69], suggesting that menstrual pain, irregularities, infections, and emotional distress may reflect not only individual experiences but also the cumulative effects of structural and social adversity. Experiences reported by LGBTI participants further suggest that gender identity and societal expectations surrounding menstruation may intersect to generate additional forms of distress. This interpretation is consistent with minority stress theory, which proposes that stigma and social marginalization experienced by minority groups can contribute to chronic psychological stress [70].

An intersectional lens is critical for understanding the complexity of menstrual health experiences during displacement [71]. In the included studies, women and girls’ experiences were shaped by intersecting dimensions of vulnerability, including gender, age, displacement status, economic conditions, and social roles. For example, adolescent girls experiencing menarche without prior knowledge were particularly vulnerable, while women with caregiving responsibilities navigated competing demands that influenced menstrual management. Consistent with previous research, these intersecting factors are associated with inequalities in menstrual health and access to resources [72,73], particularly in low-resource and humanitarian contexts [71,74]. An intersectional perspective provides a more nuanced understanding of how overlapping social and structural determinants shape menstrual experiences and can inform more equitable, context-sensitive, and inclusive interventions.

Although refugee and humanitarian settings introduce challenges related to displacement, insecurity, and resource scarcity, many of the themes identified in this meta-synthesis are consistent with findings from previous reviews in non-refugee populations [5]. This suggests that menstrual experiences are shaped across contexts by sociocultural norms, menstrual stigma, and gendered expectations. However, humanitarian conditions may amplify these processes by reducing access to resources, limiting autonomy, and increasing vulnerability, thereby intensifying their impact on menstrual health experiences and outcomes.

### 4.1. Implications for Future Research and Practice

The findings of this meta-synthesis indicate that menstrual health is not only a health issue but also a matter of dignity, social justice, and human rights. Menstrual stigma and structural barriers contribute to fear of judgment, internalised stigma, and reduced agency among refugee women and girls. Addressing these challenges therefore requires tailored, culturally sensitive, and multifaceted interventions that are both gender-responsive and gender-transformative [60,75]. Importantly, engaging men and boys as active participants in menstrual health interventions may help disrupt stigma and reshape gender norms, fostering more supportive and less discriminatory environments [76].

The mismatch between women and girls’ needs and current humanitarian responses highlights persistent programming gaps. Although interventions such as menstrual product distribution are increasingly implemented, they often remain fragmented and insufficient to address the structural and sociocultural determinants of menstrual health. In line with international guidance from the Sphere Handbook [9], UNICEF, and WHO [77,78], addressing these gaps requires integrated, multisectoral approaches involving WASH, health, education, and protection sectors. Practical tools such as the MHM Toolkit [20] may support the implementation of context-sensitive strategies, including needs assessments, provision of materials, and the development of safe and private WASH facilities.

Strengthening menstrual health literacy is a key priority. Stigma and limited access to comprehensive information may reinforce gender inequalities and contribute to adverse social outcomes, including school absenteeism [60,79]. Participatory and empowerment-based approaches, including digital and community-based strategies, may improve menstrual and reproductive health literacy [80,81]. Schools may serve as important entry points for menstrual health education [20,78], complemented by community-based initiatives that engage families, including mothers, female relatives, and male community members [82,83].

Previous evidence indicates that access to sexual and reproductive health services among refugee women remains constrained by persistent structural and sociocultural barriers [84,85]. Robust evidence on the effectiveness of interventions in these settings also remains limited. In this context, skilled health personnel, particularly midwives, may play a key role in promoting menstrual health literacy, engaging communities, and supporting integrated, rights-based approaches to sexual and reproductive health during humanitarian crises [17].

Future research should adopt intersectional and context-sensitive approaches to deepen understanding of menstrual health across different stages of the migration journey, evaluate the effectiveness of integrated interventions, and explore the experiences of underrepresented groups, including women and girls with disabilities and LGBTI individuals. Overall, recognising menstrual health as a matter of dignity and human rights is essential to advancing equitable responses that address both structural determinants and sociocultural norms through integrated and evidence-informed approaches [86].

### 4.2. Limitations

This meta-synthesis has several limitations. First, variations in study design, quality, and reporting may have influenced the interpretation and synthesis of findings. Potential overlap in participants was identified in a small number of studies. However, these studies addressed different research objectives and contributed distinct qualitative findings, thereby supporting a more comprehensive understanding of menstrual health experiences among refugee women and girls. Nevertheless, some overrepresentation of specific experiences cannot be fully excluded. Furthermore, the included studies were conducted across diverse geographical and sociocultural humanitarian contexts, spanning different time periods. Changes in humanitarian responses, sociopolitical conditions, and menstrual health awareness over time may have influenced participants’ experiences and how menstrual health challenges were conceptualised and reported. Although common patterns emerged across studies, temporal and contextual differences should be considered when interpreting the transferability of the findings.

Second, the analysis relied on published data and may not fully capture the depth and nuance of participants’ lived experiences.

Finally, the review only included studies published in English, Italian, Spanish, and Portuguese. This language restriction may have excluded relevant evidence published in other languages, particularly from regions heavily affected by forced displacement, potentially limiting the cultural and contextual diversity of the synthesis.

## 5. Conclusions

This meta-synthesis demonstrates that menstrual health among refugee women and girls is shaped by the complex interplay of structural constraints, sociocultural norms, and embodied experiences. The menstrual health challenges they face are not merely the result of individual behaviours but reflect broader systemic inequities that limit access to resources, information, and supportive environments. Despite these challenges, women and girls actively navigate constraints through adaptive strategies, often at significant cost to their health, dignity, and well-being. Addressing menstrual health in humanitarian settings therefore requires integrated, intersectional, and rights-based approaches that move beyond short-term solutions to tackle underlying determinants. Ensuring equitable access to menstrual health is essential not only for improving health outcomes, but also for promoting dignity, agency, and social justice among displaced populations. Achieving this goal requires coordinated action across humanitarian, health, education, WASH, and protection sectors, alongside the active involvement of communities, families, men and boys, and healthcare professionals, including midwives, to foster supportive environments and deliver sustainable, culturally sensitive, and evidence-informed menstrual health interventions.

## Figures and Tables

**Table 1:** Studies included in the meta-synthesis (N = 24).

Record N°	Authors (Year)	Study Design	Theoretical Perspective	Aim	City/Region (Country); Setting	Sample	Data Collection Method
1	Abuzerr et al. (2026) [42]	Qualitative, exploratory research	Feminist political ecology and intersectionality theory	To examine the gendered dimensions of WASH insecurity and its impacts on the health and well-being of IDPs	Gaza Strip Governorates (Palestine); informal displacement sites and makeshift shelters	26 internal displaced women: 18 adult women; 8 adolescent girls (13–58 years)	Semi-structured IDIs and FGDs
2	Akik et al. (2022) [48]	Mixed-methods design	Not reported	To explore the menstrual hygiene practices and the acceptability of reusable sanitary pads among refugee women and girls	Tripoli (Lebanon); primary health care centre	10 vulnerable Lebanese and Syrian refugee women	2 FGDs
3	Betsu et al. (2024) [35]	Qualitative study with phenomenological design	Phenomenology	To assess menstrual hygiene management experiences among displaced adolescent girls	Mekelle, Tigray Region (Ethiopia); camps for internally displaced people within the city	39 internal displaced adolescent girls (13–19 years)	6 IDIs and 4 FGDs
4	Crankshaw et al. (2024) [36]	Qualitative	Not reported	To explore the reproductive health and rights needs and challenges amongst young refugee women	eThekwini, KwaZulu-Natal province (South Africa)	35 young refugee women (18–24 years), including: first generation primary refugees; second generation children of refugees (born in South Africa or arrived in South Africa at a young age)	Semi-structured interviews
5	El Ayoubi et al. (2021) [43]	Qualitative descriptive	Not reported	To describe Syrian refugee adolescent girls’ access to SRH information	Bekaa governorate (Lebanon)	11 married adolescents (15–20 years, marriage before 18 y); unmarried adolescents (14–17 years): 5 to 7 participants per FGD; and mothers of girls aged 11–14 year: 4 to 8 participants per FGD	11 IDIs with married adolescents; FGDs: 3 with unmarried adolescents, and 2 with mothers
6	Ghandour et al. (2022) [44]	Qualitative	Not reported	To understand the menstrual preparedness of adolescent refugee girls	West Bank and Jordan (Palestine); Palestinian refugee camps	232 adolescent girls (mostly aged 14–18 years)	39 individual IDIs and 23 FGDs
7	Hamamra et al. (2025) [45]	Qualitative	Not reported	To explore the challenges faced by Palestinian women about menstrual health during the war	Rafah (Palestine); Palestinian IDPs camps	32 refugee women (20–53 years)	Semi-structured interviews
8	Ivanova et al. (2019) † [37]	Mixed-methods	Not reported	To assess the sexual and reproductive health experiences, knowledge and access to services among adolescent refugee girls	Isingiro District (Southwest Uganda); Nakivale refugee settlement	23 adolescent refugee girls (13–15 years)	Semi-structured interviews
9	Kemigisha et al. (2020) † [38]	Qualitative	Not reported	To describe menstruation practices and experiences of adolescent refugee girls	Isingiro District (Southwest Uganda); Nakivale refugee settlement	42 adolescent refugee girls (13–19 years)	28 semi-structured interviews and 2 FGDs
10	Korri et al. (2021) [46]	Qualitative	Not reported	To examine the SRH perceptions and experiences	Bourj Hammoud, Beirut (Lebanon)	Kurdish and Arab Syrian refugee adolescent girls 13–17 years	FGDs
11	Kulig (1988) [53]	Qualitative	Not reported	To identify cultural knowledge of conception and its relationship to use of birth control among Cambodian refugee women.	Western Canada	30 Cambodian refugee women and a crou khmer	Participant observation and ethnographic interviews
12	Logie et al. (2025) [16]	Qualitative	Not reported	To explore the impact of climate change and related EWE on SRH among refugee youth in Bidi Bidi Refugee Settlement, Uganda.	(Northwestern Uganda); Bidi Bidi Refugee Settlement	32 refugees (mean age 20 years)	Semi-structured interviews and one-on-one interviews. ‘Walk-along’ interview and ‘go-along’ methodological approaches. Multi-sensory analysis.
13	Loutet et al. (2024) [39]	Qualitative	Not reported	To understand how refugee youth living in Bidi Bidi Refugee Settlement felt towards comfort, safety, and willingness to talk about sexual health.	(Northwestern Uganda); Bidi Bidi Refugee Settlement	40 refugees aged 16–24 years	FGDs
14	Majed & Touma (2020) [49]	Qualitative and community- based participatory research approach	Not reported	To explore the different challenges related to menstrual hygiene faced by Syrian refugee women.	Beeka Valley (Lebanon); Informal Tented Settlement in 10 municipalities (Saide, Bouday, Hourtaala, Houch Barada, Houch Tal Safeyi, Deir el Ahmar, Btedie, Jabaa, Chlifa and Talia)	Over 130 female refugees aged 15–50 years (1 girl with disability)	10 FGDs and 38 in-depth semi-structured interviews
15	Pandit et al. (2022) [47]	Mixed-methods	Not reported	To review and describe menstrual hygiene management (MHM) along with the existing challenges of MHM among Rohingya adolescent girls.	Ukhiya, Cox’s Bazar (Bangladesh); Kutupalong refugee camps	Adolescents aged 13–18 years living in Kutupalong refugee camp	12 FGDs
16	Parker et al. (2014) [40]	Qualitative	Not reported	To discuss the impact of limited menstrual management resources on the well-being, education, and economic activities of women in low-income countries.	District of Katakwi (Northeast Uganda); 13 IDP camps	450 internally displaced women	10 FGDs
17	Rakhshanda et al. (2021) [21]	Mixed-methods	Not reported	To understand the knowledge and practice along with some associated factors regarding MHM among adolescent girls in selected Rohingya refugee camps	Subdistrict of Cox’s Bazar (Bangladesh); refugee settlements	340 adolescent girls (14–18 years old) and their mothers	IDIs and FGDs
18	REACH, UNICEF (2019) [50]	Qualitative assessment	Not reported	To identify WASH needs and service gaps among the Rohingya refugee population	District of Cox’s Bazar (Bangladesh); Rohingya refugees in camps in Ukhiya and Teknaf Upazilas	54 participants (25 female and 29 male)	6 FGDs
19	Schmitt et al. (2017) [13]	Qualitative	Not reported	1. To identify key barriers to MHM, and to what degree each response addressed MHM needs. 2. To generate insights into the types and content of MHM guidance that would improve coordination and response in future emergencies.	Rakhine State (Myanmar) and Tripoli, Beirut and the Bekaa Valley (Lebanon); IDP camps	17 emergency response staff; 117 woman (19–49 years); 71 adolescents (14–18 years)	Key Informant Interviews; FGDs; Participatory mapping
20	Schmitt et al. (2022) [41]	Qualitative	Not reported	To examine how displaced girls and women were managing their MHM needs, with an emphasis on menstrual disposal and laundering practices and challenges.	Maiduguri, Borno State (Nigeria); IDP camps	11 NGO workers and UN Agency Staff; 70 displaced women and adolescents.	Key Informant Interviews; FGDs; direct observations of WASH infrastructure.
21	Sherally et al. (2025) [33]	Mixed- methods	Not reported	To explore how displacement impacts the sexual and SRH of refugee women.	Lesbos (Greece); CCAC or Mavrovouni	6 refugee women	FGDs
22	UNFPA (2022) [51]	Mixed-methods	Not reported	To evaluate the knowledge, conditions and practices of refugee women and girls regarding to menstrual hygiene management and the extent of period poverty	Türkiye; UNFPA supported centres: Diyarbakir WGSS, Sanliurfa WGSS, Eskisehir WGSS, Eskisehir Youth Centre and Sanliurfa Women and Youth Health and Support Centre	32 refugee girls and women (15–45 years)	5 FGDs
23	UNFPA (2022) [52]	Mixed- methods	Not reported	To analyse the menstrual practices of LGBTI refugees, including access to menstrual products, product acceptability, and menstrual hygiene practices	Denizli, Eskişehir, Mersin, and Istanbul (Türkiye)	8 participants, including two cisgender lesbian women, one cisgender bisexual woman and five trans heterosexual men (19–45 years), coming from Iran, Jordan, Morocco, and Iraq	2 FGDs and 2 key informant interviews
24	VanLeeuwen & Torondel (2018) [34]	Qualitative	Not reported	To explore the acceptability and utility of menstrual underwear among the female refugee population.	Ritsona, rural area (Greece); Ritsona refugee site	30 refugee women (18–50 years); 5 female humanitarian staff	11 semi-structured interviews and 21 FGDs

Notes. WASH: Water, Sanitation, and Hygiene; IDPs: Internally Displaced Persons; IDIs: In-Depth Interviews; FGDs: Focus Group Discussions; MHM: Menstrual Health Management; SRH: Sexual and Reproductive Health; CCAC: Closed Controlled Access Centre; NGO: non-governmental organization; EWE: extreme weather events; UNICEF: United Nations International Children’s Emergency Fund; UNFPA: United Nations Population Fund; WGSS: Women and Girls’ Safe Spaces. † Potential overlap in sample and setting between Ivanova et al. (2019) [37] and Kemigisha et al. (2020) [38].

**Table 2:** Quality appraisal of included studies (JBI checklist) [20].

Study (Author, Year)	1	2	3	4	5	6	7	8	9	10	Inclusion
Abuzerr et al., 2026 [42]	Y	Y	Y	U	Y	N	U	Y	Y	Y	Included
Akik et al., 2022 [48]	NA	Y	Y	U	Y	N	N	Y	U	Y	Included
Betsu et al., 2024 [35]	NA	Y	Y	Y	Y	Y	N	Y	Y	Y	Included
Crankshaw et al., 2024 [36]	NA	Y	Y	Y	Y	Y	N	Y	Y	U	Included
El Ayoubi et al., 2021 [43]	NA	Y	Y	Y	Y	Y	Y	Y	Y	Y	Included
Ghandour et al., 2026 [44]	NA	Y	Y	Y	Y	Y	U	Y	Y	Y	Included
Hamamra et al., 2025 [45]	NA	Y	Y	Y	Y	Y	Y	Y	Y	Y	Included
Ivanova et al., 2019 [37]	NA	Y	Y	Y	Y	N	N	Y	Y	Y	Included
Kemigisha et al., 2020 [38]	NA	Y	Y	Y	Y	N	N	Y	Y	Y	Included
Korri et al., 2021 [46]	Y	Y	Y	Y	Y	Y	Y	Y	Y	Y	Included
Kulig, 1988 [53]	NA	Y	Y	Y	U	N	Y	Y	N	Y	Included
Logie et al., 2025 [16]	NA	Y	Y	Y	Y	N	U	Y	Y	Y	Included
Loutet et al., 2024 [39]	NA	Y	Y	Y	Y	Y	U	Y	Y	Y	Included
Majed & Touma, 2020 [49]	NA	Y	Y	U	U	N	N	Y	Y	Y	Included
Pandit et al., 2022 [47]	NA	Y	Y	Y	Y	Y	U	Y	Y	Y	Included
Parker et al., 2014 [40]	Y	Y	Y	Y	Y	Y	U	Y	N	Y	Included
Rakhshanda et al., 2021 [21]	NA	Y	Y	Y	Y	N	U	Y	Y	Y	Included
REACH, UNICEF, 2019 [50]	NA	Y	Y	U	Y	N	N	Y	Y	Y	Included
Schmitt et al., 2017 [13]	NA	Y	Y	Y	Y	U	U	Y	N	Y	Included
Schmitt et al., 2022 [41]	NA	Y	Y	Y	Y	N	N	Y	Y	Y	Included
Sherally et al., 2025 [33]	NA	Y	Y	Y	Y	N	U	Y	Y	Y	Included
Turkmen Sanduvac et al., 2017 [32]	NA	Y	U	N	N	N	N	U	N	N	Excluded
UNFPA, 2022 [51]	NA	Y	Y	U	Y	N	N	Y	U	Y	Included
UNFPA, 2022 [52]	NA	Y	Y	U	Y	N	N	Y	U	Y	Included
Van Leeuwen & Torondel, 2018 [34]	NA	Y	Y	Y	Y	N	N	Y	Y	Y	Included

Y = Yes; N = No; U = Unclear; NA = Not Applicable. 1. Congruence of philosophical perspective/methodology; 2. Congruence of methodology/objectives; 3. Congruence of methodology/data collection; 4. Congruence of methodology/data analysis; 5. Congruence of methodology/interpretation of results; 6. Cultural and theoretical context of the researcher; 7. Influence of the researcher on the research; 8. Participants represented; 9. Research ethics committee approval; 10. Conclusions from data analysis/interpretation.

**Table 3:** Codes, descriptive themes, and analytical themes derived from the thematic synthesis.

Analytical Theme	Descriptive Theme	Code
Theme 1: Structural constraints shaping access to menstrual health resources and services	1.1 Environmental and infrastructural barriers	Lack of access to water, Lack of lighting, Inadequate sanitation facilities, Unsafe environment, Environmental conditions, Coping strategies in response to lack of privacy in sanitation facilities
	1.2 Economic constraints and limited access to menstrual and hygiene resources	Limited availability of menstrual and hygiene products, Economic constraints, Competing basic needs and resource scarcity, Distribution of menstrual hygiene products, Availability of menstrual products through local markets, Resource-constrained coping strategies
	1.3 Access to and quality of healthcare services for menstrual health	Awareness and knowledge, Access and availability, Barriers to healthcare-seeking behaviours, Inadequate quality of care, Positive experiences with healthcare
Theme 2: Embodied and context-dependent menstrual management practices	2.1 Menstrual management practices	Menstrual materials: Disposable menstrual pads, Reusable cloth pads, Menstrual underwear
		Menstrual management practices: Changing menstrual materials, Disposal of menstrual materials, Washing and drying clothing pads, Transport of menstrual materials, Hygiene-related practices, Adaptive/coping strategies: using improvised materials
		Contextual constraints shaping practices: Menstruation management during night, Menstruation in transit, Menstruation before the displacement
	2.2 Experiences of menarche and menstrual preparedness	Menarche experiences, Preparedness for menarche, Maternal presence and support during menarche
Theme 3: Female support networks as a key resource in menstrual management	3.1 Female social and familial support networks in menstrual management	Maternal support, Support from female family members, Peer-to-peer support, Informal female support networks
Theme 4: Menstruation as a socially constructed and learned experience	4.1 Transmission of knowledge and knowledge gaps	Knowledge and knowledge gaps: Lack of knowledge, Adequate menstrual knowledge
		Sources of menstrual transmission of knowledge: Education at school, Education provided by mothers, Education provided by family members, Peer-to-peer knowledge transmission, Other sources of menstrual information
		Characteristics of knowledge transmission: Education shaped by fear and restrictions, Education on religious and social norms
	4.2 Cultural beliefs and practices about menstruation	Cultural beliefs about menstruation, Misconceptions and myths, Cultural practices during menstruation, Menstrual taboo, Menstruation not considered taboo
		Perceptions of menstruation and menstrual blood: Negative perceptions of menstrual blood, Positive perception of menstruation, Normalization of menstruation
Theme 5: Constrained agency and compromised dignity under conditions of menstrual stigma and exposure	5.1 Menstrual stigma and social exposure	Lack of privacy, Forced exposure, Fear of being seen, Fear of leakage, Hiding menstruation, Perceived stigma and social judgement
	5.2 Restricted agency and loss of dignity in menstrual management	Inability to act, Lack of control, Loss of dignity, Perceived neglect and low prioritization of menstrual health needs, Enduring without choice, Restricted participation in daily activities
Theme 6: Physical and psychological impacts of menstrual health challenges	6.1 Physical well-being and health	Health problems, Infections, Physical discomfort, Menstrual pain
	6.2 Emotional and psychological burden	Emotional distress and negative affect: Anxiety, Emotional distress, Fear, Fear for personal safety, Confusion, Social and emotional discomfort
		Shame and stigma-related emotions: Shame, Gender-related distress

**Table 4:** Matrix of studies and analytical themes.

Authors (Year)	Theme 1	Theme 2	Theme 3	Theme 4	Theme 5	Theme 6
Abuzerr et al., 2026 [42]	x	x	x	-	x	x
Akik et al., 2022 [48]	x	x	-	-	x	x
Betsu et al., 2024 [35]	x	x	-	x	x	x
Crankshaw et al., 2024 [36]	-	x	x	x	-	x
El Ayoubi et al., 2021 [43]	-	x	x	x	x	x
Ghandour et al., 2026 [44]	x	x	x	x	x	x
Hamamra et al., 2025 [45]	x	x	x	x	x	x
Ivanova et al., 2019 [37]	x	x	x	x	x	x
Kemigisha et al., 2020 [38]	x	x	x	x	x	x
Korri et al., 2021 [46]	x	x	x	x	-	x
Kulig, 1988 [53]	-	x	-	x	-	x
Logie et al., 2025 [16]	x	-	-	x	x	x
Loutet et al., 2024 [39]	x	x	-	x	-	x
Majed & Touma, 2020 [49]	x	x	x	x	x	x
Pandit et al., 2022 [47]	x	x	x	x	x	x
Parker et al., 2014 [40]	x	x	x	x	x	x
Rakhshanda et al., 2021 [21]	x	x	x	x	x	x
REACH, UNICEF, 2019 [50]	x	x	-	x	x	x
Schmitt et al., 2017 [13]	x	x	-	x	x	x
Schmitt et al., 2022 [41]	x	x	-	x	x	x
Sherally et al., 2025 [33]	x	x	x	-	-	x
UNFPA, 2022 [51]	x	x	x	x	x	x
UNFPA, 2022 [52]	x	x	-	x	x	x
Van Leeuwen & Torondel, 2018 [34]	x	x	x	x	x	x

Note: ‘x’ indicates that the analytical theme was identified in the study; ‘-‘ indicates that the analytical theme was not identified.

## Data Availability

No new data were created. All data supporting the findings of this study are derived from publicly available published studies cited in this article. Extracted data and analytic materials (e.g., coding framework) are available from the corresponding author upon reasonable request.

## Supplementary Materials

The following supporting information can be downloaded at https://www.mdpi.com/article/10.3390/healthcare14131974/s1: Table S1: ENTREQ Checklist; Table S2: PRISMA Checklist.

## Abbreviations

The following abbreviations are used in this manuscript:

SRH	Sexual and Reproductive Health
SDG	Sustainable Development Goals
WASH	Water, Sanitation, and Hygiene
IDPs	Internally Displaced Persons
FGDs	Focus Group Discussions
NGO	Non-Governmental Organization
MHM	Menstrual Health Management
FGM	Female Genital Mutilation

## Appendix A

**Table A1:** Research question (S.P.I.D.E.R.).

Sample	Phenomenon of Interest	Design	Evaluation	Research Type
Women and girls: - Refugees - Asylum seekers - Forced displacement - Irregular migrants - Reproductive age (including menarche) - At any stage of the migration and reception process (transit, stay in refugee camps, reception centres, informal settlements)	Lived experience of menstrual health during migration, including: - Subjective experience of menstruation - Experience of menarche in a migratory context - Cultural meanings attributed to menstruation - Emotional and psychological experiences - Menstrual management practices - Barriers to menstrual health management - Facilitators and resources - Coping and empowerment strategies	Qualitative or qualitative studies, including: - Individual interviews (in-depth, semi-structured) - Focus groups - Ethnography - Phenomenological studies - Grounded theory - Narrative inquiry - Mixed studies with separately analysable qualitative data	Themes, perceptions, meanings, representations, experiences, barriers, facilitators, perceived needs	- Qualitative studies - Mixed-methods study - Qualitative research reports published in peer-reviewed journals

**Table A2:** Search strategy for electronic databases.

Database	Search String	Filters	Records
PubMed	*((((“Transients and Migrants”[Mesh] OR migrant*) OR (“Emigrants and Immigrants”[Mesh] OR emigrant* OR immigrant*) OR (“Refugees”[Mesh] OR refugee* OR “Asylum seeker*” OR “Displaced person*” OR “Internally displaced person*” OR “IDP*” OR “Forcibly displaced person*”)) OR (“Human Migration”[Mesh] OR “human migration*” OR immigration* OR emigration*)) AND ((((“Menstruation”[Mesh] OR menstruat* OR menses) OR (“Menstrual Cycle”[Mesh] OR “menstrual cycle” OR menstrual* OR menstruum OR “menstrual period*”)) OR (“Menarche”[Mesh] OR menarche)) OR (“Menstrual Hygiene Products”[Mesh] OR “menstrual hygiene product*” OR “menstrual hygiene” OR “menstrual health” OR “menstrual product*” OR “menstrual tampon*” OR “vaginal tampon*” OR “menstrual pad*” OR “feminine napkin*” OR “menstrual napkin*” OR “menstrual cup*”))) AND ((((((((((“Personal Narrative” [Publication Type] OR “personal narrative*” OR narrative* OR narration* OR perception* OR sentiment* OR opinion* OR beliefs OR meaning* OR perspective* OR “point of view*” OR viewpoint* OR experience* OR “lived experience*”) OR (“Health Knowledge, Attitudes, Practice”[Mesh] OR “knowledge, attitude*, practice*”)) OR (“Attitude to Health”[Mesh] OR “attitude* to health” OR attitude*)) OR (“Social Stigma”[Mesh] OR “social stigma*” OR stigma*)) OR (“Shame”[Mesh] OR shame*)) OR (“Confidentiality”[Mesh] OR secrecy)) OR (“Taboo”[Mesh] OR taboo*)) OR (“Coping Skills”[Mesh] OR “coping skill*” OR coping*)) OR (“Superstitions”[Mesh] OR superstition* OR embodiment*)) OR (“Needs Assessment”[Mesh] OR “health need*” OR “unmet need*” OR barrier* OR facilitator* OR “barrier* and facilitator*”))*	Language (English, Italian, Spanish, Portuguese)	123 (15 March 2026)
Cochrane Library	*migrant OR emigrant OR immigrant OR refugee OR “Asylum seeker” OR “Displaced person” OR “Internally displaced person” OR “IDP” OR “Forcibly displaced person” OR “human migration” OR immigration OR emigration* *AND* *menstruation OR menstruate OR menses OR “menstrual cycle” OR menstrual OR menstruum OR “menstrual period” OR menarche OR “menstrual hygiene product” OR “menstrual hygiene” OR “menstrual health” OR “menstrual product” OR “menstrual tampon” OR “vaginal tampon” OR “menstrual pad” OR “feminine napkin” OR “menstrual napkin” OR “menstrual cup”* *AND* *“personal narrative” OR narrative OR narration OR perception OR sentiment OR opinion OR beliefs OR meaning OR perspective OR “point of view” OR viewpoint OR experience OR “lived experience” OR “knowledge, attitude, practice” OR “attitude to health” OR attitude OR “social stigma” OR stigma OR shame OR secrecy OR taboo OR “coping skill” OR coping OR superstition OR embodiment OR “health need” OR “unmet need” OR barrier OR facilitator OR “barrier and facilitator”*	/	2 (13 March 2026)
Scopus	*migrant* OR emigrant* OR immigrant* OR refugee* OR “Asylum seeker*” OR “Displaced person*” OR “Internally displaced person*” OR “IDP*” OR “Forcibly displaced person*” OR “human migration*” OR immigration* OR emigration** *AND* *menstruat* OR menses OR “menstrual cycle” OR menstrual* OR menstruum OR “menstrual period*” OR menarche OR “menstrual hygiene product*” OR “menstrual hygiene” OR “menstrual health” OR “menstrual product*” OR “menstrual tampon*” OR “vaginal tampon*” OR “menstrual pad*” OR “feminine napkin*” OR “menstrual napkin*” OR “menstrual cup*”* *AND* *“personal narrative*” OR narrative* OR narration* OR perception* OR sentiment* OR opinion* OR beliefs OR meaning* OR perspective* OR “point of view*” OR viewpoint* OR experience* OR “lived experience*” OR “knowledge, attitude*, practice*” OR “attitude* to health” OR attitude* OR “social stigma*” OR stigma* OR shame* OR secrecy OR taboo* OR “coping skill*” OR coping* OR superstition* OR embodiment* OR “health need*” OR “unmet need*” OR barrier* OR facilitator* OR “barrier* and facilitator*”*	Language (English, Italian, Spanish, Portuguese)	185 (15 March 2026)

**Table A3:** Search strategy for grey literature.

Source (Website)	Search String	Filters	Records
ReliefWeb (https://reliefweb.int)	(menstruation OR menstrual) AND refugee	Content format (Analysis, Assessment, Evaluation and Lesson Learned, UN Document, Other)	89
United Nations High Commissioner for Refugees (UNHCR) (https://www.unhcr.org)	menstruation AND refugee AND qualitative	Publication category (Data and Statistics, Reports)	6
United Nations International Children’s Emergency Fund (UNICEF) (https://www.unicef.org)	menstruation AND refugee AND qualitative	Type of content (Annual report, Document, Flagship report, Report, Situation report)	75
World Health Organization (WHO) (https://www.who.int)	menstruation AND refugee AND experience	/	28
WHO/UNICEF Joint Monitoring Programme for Water Supply, Sanitation and Hygiene (JMP) (https://washdata.org)	menstruation OR menstrual	/	2
United Nations Population Fund (UNFPA) (https://www.unfpa.org)	menstruation AND refugee AND experience	/	13
International Organization for Migration (IOM) (https://www.iom.int)	menstrual health OR menstruation	/	No results
Médecins Sans Frontières (MSF) (https://www.msf.org)	menstruation OR menstrual	/	8
Save the Children (STC) (https://www.savethechildren.net)	menstruation OR menstrual	/	18
Oxford Committee for Famine Relief (OXFAM) (https://policy-practice.oxfam.org)	menstruation OR menstrual	/	13
International Rescue Committee (IRC) (https://www.rescue.org)	menstruation OR menstrual	/	No results
Cooperative for Assistance and Relief Everywhere (CARE) International (https://www.care-international.org)	menstruation OR menstrual	/	No results

Note. All websites were accessed on 29 May 2026.
